# The role of hazard vulnerability assessments in disaster preparedness and prevention in China

**DOI:** 10.1186/s40779-015-0059-9

**Published:** 2015-11-24

**Authors:** Yan Du, Yibo Ding, Zixiong Li, Guangwen Cao

**Affiliations:** Department of Epidemiology, Second Military Medical University, 800 Xiangyin Road, Shanghai, 200433 People’s Republic of China

**Keywords:** Vulnerability, Hazard vulnerability assessment, Disaster epidemiology, Disaster preparedness

## Abstract

China is prone to disasters and escalating disaster losses. Effective disaster mitigation is the foundation for efficient disaster response and rescue and for reducing the degree of hazardous impacts on the population. Vulnerability refers to the population’s capacity to anticipate, cope with, and recover from the impact of a hazardous event. A hazard vulnerability assessment (HVA) systematically evaluates the damage that could be caused by a potential disaster, the severity of the impact, and the available medical resources during a disaster to reduce population vulnerability and increase the capacity to cope with disasters. In this article, we summarized HVA team membership, content (disaster identification, probability and consequences), and methods and procedures for an HVA that can be tailored to China’s needs. We further discussed the role of epidemiology in an HVA. Disaster epidemiology studies the underlying causes of disasters to achieve effective disaster prevention and reduction. In addition, we made several recommendations that are already in practice in developed countries, such as the U.S., for future implementation in China and other developing countries. An effective HVA plan is crucial for successful disaster preparedness, response, and recovery.

## Background

Disasters occur frequently and often place a substantial burden on affected populations. Disasters are defined as singular large-scale events that cause serious disruptions of the function of a community or a society and involve human, material, economic or environmental losses or impacts. Those losses or impacts often exceed the community’s or society’s ability to control or cope with the disaster using its existing resources. Disasters are the product of a combination of hazards and vulnerability. Hazards that strike in areas with low vulnerability will not become disasters [1]. However, most hazards occur in developing or underdeveloped regions or in areas with a high population density, poor infrastructure, and a limited or no disaster preparedness plan. Comparing to developed countries, such as the U.S., where emergency planning and other disaster-related fields have been extensively studied and implemented, disaster-related fields such as disaster medicine education and research are fairly new in China. There is a significant gap between the knowledge level of and skills for disaster medicine and the current needs of disaster preparedness [2]. High vulnerability and insufficient disaster preparedness plans have severe consequences. For example, during the 2003 SARS outbreak, without an effective HVA on emerging infectious diseases, some Chinese hospitals could not effectively response to this severe infectious disease and fell into crisis. More recently, the Eastern Star yacht, which was carrying more than 450 people, sank overnight in the Yangtze River during a cyclone in southern China, and only eight people were rescued. The causes were extreme weather conditions and possible human error.

It is important for China to learn about successful experiences and current best practices and to integrate available resources to set up systematic and functional disaster preparedness plans. In this article, we focused on the hazard vulnerability assessment aspect, which is almost nonexistent in China, in relation to disaster preparedness. We briefly summarized and discussed the current experiences and information that can be tailored to China’s needs. In combination with our own work in disaster medicine, we emphasized the epidemiological component of the hazard vulnerability assessment.

## Vulnerability

Vulnerability is defined as “the characteristics of a person or group and their situation that influences their capacity to anticipate, cope with, resist, and recover from the impact of a hazardous event” [3]. Vulnerability represents the susceptibility of a given population to harmful effects from exposure to hazardous events. It directly affects disaster preparation, response, and recovery [4]. Hazardous event can directly or indirectly affect the health status of an individual or a population. There are two key components of a hazard’s definition: the extent of the influence (harm) caused by the exposure to the disaster and the “differential” sensitivity to and ability to recover from the disaster. Different populations have different levels of vulnerability to a hazardous event that depend on multiple uncertain factors, including hazard intensity, environment, and population characteristics [5]. Hazard intensity is exposure related, which is directly related to the occurrence of natural disasters, such as heavy rainfall that may cause floods and/or landslides. Environmental and population characteristics are system-resistance factors related to population vulnerability level. Environmental factors can amplify or mitigate the destructive power of a hazardous event. For example, good water-soil conservation capabilities can reduce the effects of a mudslide. Social vulnerabilities are related to environmental and population characteristics, which are influenced by exposure, sensitivity, and resilience. Exposure is related to hazard proximity and the environmental characteristics [6]. Sensitivity refers to the ability of an individual or community to protect itself from different types of potential harm, while resilience indicates an individual’s or community’s coping and adaptive capacities during and after a disaster [6]. Vulnerable populations usually include those with low incomes; individuals who may be chronically or terminally ill, physically or mentally disabled, homeless, or uninsured or underinsured; and the elderly, children, and pregnant women. One ongoing area of vulnerability is the surge capacity for large-scale events. During the acute response phase, which is the first 96 h after a disaster occurs, psychological sequelae coupled with stress may catapult some preconditions into full-blown illnesses that need immediate and specialized medical services. In addition, the health impact of the disaster may cause an increased or fluctuating demand for health care services throughout the long-term recovery period, which is the secondary surge capacity [7]. In addition to the physical needs of the vulnerable population, the serious and widespread mental health consequences of disasters should receive sufficient attention. At least some members of the vulnerable population will need post-disaster psychological support, especially in low- and middle-income countries where mental health services are scarce [8]. For example, the prevalence of post-traumatic stress disorder (PTSD) among survivors remained relatively common even 5 years after China’s Wenchuan earthquake (9.2 % in 2013), and the burden was especially high for females, farmers, elderly survivors and those who lost family members [9].

Policies aimed at disaster prevention and rescue must take into consideration the hazard intensity, environmental, and population characteristics that will affect the impact and outcomes of a disaster. The changes of a population’s living environment will influence the population’s “absorbing” ability regarding the damage. The factors affecting the “absorbing” ability are generally classified into two categories. One is at the community level and includes such factors as population growth and distribution, especially increased population density and urbanization, deforestation, dense infrastructure, traffic congestion, river dredging, hospital distribution, socioeconomic status, occupation, and daily activity patterns and habitats. For example, with the rapid urbanization in China, the population is heavily weighted toward the eastern part of the country, which leads to a series of issues that include insufficient public health resources and infrastructure. The second category is at the national and international levels and includes such factors as global climate change, international debt relief policy, domestic land development plan, infrastructure of transportation and communication, government stability, execution of laws, public health policies, and population education level. All of these factors can be used to assess population vulnerability.

### Hazard vulnerability assessment

Population hazard vulnerability analysis has been extensively studied and proven to be effective. A hazard vulnerability assessment (HVA) is a systematic approach to identify all possible hazards that may affect a specific population, assess the risk associated with each hazard (e.g., the probability of hazard occurrence and the consequences for the population), and study the findings to develop a prioritized comparison of hazard vulnerabilities. The consequence, or vulnerability, is related to both the impact on the population and the likely service demands created by the impact [10]. An HVA can be carried out at the community level or at the hospital (and other health care facility) level. After the events of September 11, 2001, in New York, U.S., experts suggested that hospitals should function as “an integrated entity within the scope of the broader community”. Because hospitals and other health care facilities are always at the forefront to prepare for and respond to disastrous events, they are now expected to be community organizations instead of standalone institutions. We usually rely on hospitals to treat disaster victims, provide ongoing health care to the community, and prevent secondary disease outbreaks caused by the loss of infrastructure and/or sanitation [11]. An HVA is the key step in the emergency response to a disaster. It should be able to methodologically evaluate the degree of impact and provide background information to create a targeted disaster mitigation plan. An HVA can also be applied in response to manmade disasters, such as terrorist attacks. In addition, a community HVA and a hospital HVA should be designed to complement one another for disaster emergency response and rescue.

#### HVA team membership

Hospitals should develop specific plans to respond to their identified top hazards, which are based on community characteristics. Sometimes hospital and community HVAs are developed together. An HVA requires an in-depth understanding of the local community’s characteristics and is usually developed by a multidisciplinary team based on the following aspects: (1) the top hazards confronting the community; (2) the current available resources, the resources needed that are not available, and the period of time needed for the local government to acquire these resources; and (3) mitigating actions that may be applied to reduce foreseeable vulnerabilities [12].

A team approach should be adopted for the community HVA. Prospective members of the HVA team may include representatives from the emergency management agency; community leadership; community public safety agencies (police department, fire department, and emergency medical services); hospitals and other community health care facilities; public health agencies; professional groups (epidemiologists, public health workers); special hazard occupations or operations (military bases, industrial complexes, dams, and nuclear power plants); major business entities; and other emergency management planners from local, regional, or provincial agencies or private industry [13].

The hospital HVA team should also be composed of a multidisciplinary team, including representatives from at least the following areas: emergency management; security/safety; facilities (e.g., engineering, maintenance, information technology, and telecommunications); medical personnel (clinicians, nurses, laboratory staff, and radiologists); ancillary services (material, food, housekeeping, and environmental services); administrators; and finance personnel. Other stakeholders, such as representatives from the local emergency management office, fire officials, police officials, and the community, could also provide valuable input. These stakeholders are very helpful in terms of future mutual aid agreements, collaborations, and coordinated resource sharing. However, the group size should remain manageable, and regular meetings should be scheduled to discuss the maintenance of and assignments for the HVA team [13].

In addition, the military is always at the forefront of a disaster response. When disaster strikes, rapid, coordinated and appropriate responses are needed to mitigate the crisis and ensure the effective delivery of relief and aid. Military forces have the manpower, equipment, training and organization for an immediate and effective disaster response. Humanitarian relief has become a core task for defense forces. For example, the Chinese military played a key role in the emergency response and disaster relief during the Wenchuan earthquake [14]. After the earthquake, the Chinese army dispatched 215 medical, epidemic prevention, psychological assistance, and field medical equipment maintenance teams and 7000 servicemen. The military medical teams and their temporary military field hospital shelters played crucial role in medical rescue and patient treatment in the disaster zone [15]. During the 2014 Ebola outbreak in West Africa, countries such as the U.S., U.K., and China all responded promptly. The U.S. and U.K. sent troops to establish isolation units and treatment facilities, and China also dispatched several military health units to set up hospitals and testing labs [16]. Coordination between civilians and military personnel is essential, and simulations and training must be conducted on a regular basis. Military forces should coordinate with civilian authorities, such as the police, fire services and first aid providers, to set up disaster preparedness plans.

#### HVA content

Different regions and countries have different priorities for HVA content. The HVA team should first use brainstorming to determine the potential disasters that the community and/or hospitals may encounter. Disasters can be generally classified as either natural disasters or manmade disasters. Climate change and increased urbanization promote the population vulnerability to natural hazards. China is prone to a high frequency of natural disasters because of its complex geography and climate. Data show that the main disaster types include floods, earthquakes, drought, epidemics, extreme temperatures, storms, and mass movement wet (subsidence, rock falls, avalanches and landslides) [17]. These natural disasters harm humans, lead to significant economic losses, change the environment, and restrict social development. A flood is a common natural disaster that greatly affects China, dating back to 30 BC. The Yangtze River Delta region is a typical floodplain. The area’s geographic features, including numerous rivers, lakes, channels, and a low and flat terrain, make it very susceptible to multiple disasters, such as floods, typhoons, and storm surges. With a high population density and growing economy, the Yangtze River Delta region is at high risk for and very susceptible to natural hazards [18]. Earthquakes are another common disaster in China and lead to significant numbers of causalities. Three of the world’s top 10 most fatal earthquakes occurred in China, including the 1556 Shaanxi earthquake, the 1976 Tangshan earthquake, and the 1920 Haiyuan earthquake. The most recent significant earthquakes were the Wenchuan earthquake of 8 M_w_ on May 12, 2008, and the 6.6 M_w_ Lushan earthquake on April 20, 2013, which resulted in significant causalities and property losses [19]. On the other hand, increasing population density and a lack of effective prevention and response plans also increase the population vulnerability to manmade disasters, which include terrorist attacks and technological hazards. A stampede in Shanghai during the 2015 New Year’s Eve celebration left 36 dead and 47 injured, exposing issues in carrying out a proper disaster response plan [20]. A detailed database of disasters is the basis for informed policy decisions. A reliable source of information on past disasters is crucial for HVA analysis. Information can be gathered from various sources, including local residents and archives. However, objective data, such as those from academic institutions, disaster and emergency management centers, and meteorology and seismology agencies, should be used as the main references during the assessment of vulnerability. In addition, we should take into consideration secondary hazards to reduce the underestimation of population vulnerability. For example, a significant earthquake could result in various hazardous events, such as fuel shortages, transportation failures, steam failures and floods.

##### Disaster identification

Hazard identification is an important step in an HVA. It includes determining the likelihood of a disaster occurring, its intensity and magnitude, and the possible affected areas of a community [4]. Hospitals should conduct an annual review of their HVA. Extensive reviews have been conducted regarding potential threats and are summarized in Table 1.

**Table 1:** Categories and examples of potential hazards

Category	Examples
Natural disaster	Earthquake, flood, temperature extremes, hurricane (cyclone, typhoon), landslide, mudslide, severe thunderstorm, subsidence, tornado, tsunami, volcanic eruption, wildfire, windstorm, winter storm (blizzard, ice storm), epidemic
Accident	Food poisoning, stampede, road accident, aviation accident, railway accident
Hazardous material	Chemical, radiological and nuclear exposures
Technological hazard	Internal fires; fuel shortages; and potential failures involving transportation, communications, electricity, fire alarms, generators, information systems, sewage, and water

The HVA team should also evaluate the possible impacts of a disaster on the normal functions of the hospital or community. Effects caused by disasters include: (1) Human impact, such as the safety of patients, staff, and visiting family members and friends. For example, according to the Chinese national earthquake relief headquarters, after the 2008 Wenchuan earthquake, 69,227 people were killed, 374,643 were injured, 17,923 were missing, and millions were affected [15]. (2) Property impact, including but not limited to building or structure damage, loss of or damage to equipment and/or supplies, and repair costs. The 2008 Wenchuan earthquake revealed that building standards for earthquake resistance were generally poor, especially for schools and hospitals. Emergency shelters were rare in most cities [15]. (3) Business impact, such as a partial or complete business interruption, patients unable to reach the facility, employees unable or unwilling to report for work, and issues related to medical records.

##### Disaster probability and consequences

In addition to type of disaster (e.g., natural, manmade, or technological), the impact of a particular hazardous event is also determined by likelihood (probability of occurrence or frequency), severity (magnitude and intensity), and population resilience (defined as “a measure to determine the impact of available public health, emergency management, and governmental and societal resources and capabilities that could potentially mitigate negative population health consequences”) [4]. Although no indicator can accurately predict the occurrence of disasters, the combination of different risk factors can help to predict the likelihood of disaster occurrence. The likelihood of disaster occurrence can be described as low, medium, or high. Other related factors that can be used to describe the probability include: (1) the frequency of the occurrence of disaster-associated factors. For example, frequent and heavy rainfall indicates a high probability of floods; (2) the location of the hazardous event. The closer the distance between the hazard and the community, the more severe the consequence may be to the nearby community; and (3) seasonal or cyclical variations. The consequences of a disaster on the community mainly include three impacts: human, property, and business [13].

#### HVA methods

The assessment of hazard vulnerability is very helpful in emergency management. An emergency management geographic information system (GIS), including a vulnerability assessment, has been developed and applied successfully. Using socioeconomic and environmental data sources in the GIS, risk maps highlighting the potential impact of disasters on people and infrastructure can be developed that can provide guidance in terms of resource allocation and recovery operations. For example, the U.S. Federal Emergency Management Agency (FEMA) has coordinated and conducted vulnerability assessments, particularly in the area of flood mapping. Maplecroft, A UK-based global risk and strategic consulting firm, developed an index-based map to show vulnerability to climate change. The United Nations has collaborated with other entities to develop hazard risk maps for countries that are likely to face future disasters [21]. However, most emergency management GISs are designed for reaction, not prediction and mitigation. In China, to respond to a wide range of natural disasters and to reduce disaster losses, models have been developed to conduct assessments of social vulnerability to natural hazards. A data envelopment analysis (DEA)-based model was used to evaluate the relative severity of disasters in each region of mainland China [22]. Another method, which was based on the projection pursuit cluster (PPC) model, was conducted to assess social vulnerabilities to natural disasters in the Yangtze River Delta region of China [18]. More studies are needed to validate and calibrate these models. Future studies about the changes in regional vulnerability and its related causes are also of practical value.

Several toolkits have been developed to conduct HVAs. A widely used one is the Medical Center Hazard and Vulnerability Analysis, developed by Kaiser Permanente (KP) [23]. The KP HVA allows for a quantitative assessment of probability; impact (human, property, and business); preparedness; response (internal and external); and risk for different hazard categories. The degree of each measure is usually expressed as a numerical score, with 0 = N/A (non-existent), 1 = Low, 2 = Moderate, and 3 = High. Each measure is rated for separate hazardous events and weighted in the final average vulnerability score [24]. The KP HVA toolkit provides a common ground for data sharing and comparing and for prioritizing different hazards. Studies utilizing the KP HVA toolkits have been conducted to generate assessment reports of the health care systems in the United States and Abu Dhabi and provided valuable information for future HVAs [23, 25]. Another private health care product and service company, HCPro, Inc., developed its own HVA toolkit for hospitals [26]. The U.S. (FEMA Risk Analysis Branch has put considerable effort into HVA assessment and developed toolkits such as Hazus-MS [27]. Despite these efforts, none of the tools have discussed standardized procedures for HVA data collection or were designed for hospitals or communities. Hospitals are left to develop their own procedures to collect information on probability and severity, analyze the data, and interpret the results.

The second World Conference on Disaster Reduction in 1999 emphasized the change from a response culture to a prevention culture to better cope with natural disasters. Pre-disaster prevention and preparation play key roles in casualty reduction. The periodic evaluation of hospital vulnerability can help determine the impact of internal and external environment changes on hospital emergency rescue capabilities and guide disaster risk mitigation, preparedness, and recovery. An effective HVA should generate recommendations for improvement after observation and evaluation by the panel experts. Regardless of geographical, cultural, and infrastructure differences, an effective disaster management plan reduces the harmful effects of disasters on hospital emergency rescue capabilities and enables the allocation of medical resources based on the probability of disaster occurrences. For example, hospitals in the south coastal areas of China should be prepared for typhoons during the summer, while hospitals in northern China should have routine preparations for injuries or other adverse outcomes related to storms and extreme cold weather during the winter. The purpose of optimizing the allocation of hospital resources is to best utilize limited medical resources to rescue more people, which is the core value of disaster medicine. During the Wenchuan earthquake, the Chinese government established a four-level emergency response system that linked the emergency systems at the national, provincial, city, and county levels. This system coordinated the use of local resources, ensured cooperation, and enabled the effective deployment of medical rescue forces in the 2010 Yushu earthquake [15].

### The role of epidemiology in HVA

An HVA is an important component of disaster epidemiology. As stated by LeChat in 1979, “Studies on the health effects of disasters could show that epidemiological indices can be of value in planning preventive and relief measures and in evaluating their effectiveness” [28]. Disaster epidemiology is a relatively new field of study and is defined as the epidemiologic investigation of disaster forecasting and warning, emergency responses according to the different phases of disaster, and the short- and long-term adverse health effects of disasters on the population. There are two main aspects of disaster epidemiology. The first is the typical epidemiologic study of the underlying causes of disasters in order to develop prevention strategies. This aspect focuses on the disaster itself, or the mortality and morbidity associated with the disaster. The second is to investigate mechanisms to alleviate the burden of the disaster once it occurs and is usually applied at the stages of disaster preparedness or relief. A classic example is establishing functionally complete surveillance systems to monitor the possible emergence of infectious diseases. The prompt qualitative analytic assessment of different infectious disease risks after the Wenchuan earthquake improved the specificity and completeness of post-disaster disease surveillance and effectively prevented an infectious disease outbreak [15]. In addition, a mobile-phone reporting system was also proven to be successful for filing information and reporting disease numbers [15]. On the contrary, due to the low level of economic development, the lack of health resources, and the epidemic of emerging and re-emerging infectious diseases in developing countries, disasters in these countries tend to have more catastrophic damage. When the degree of disaster exceeds the “absorbing” capabilities of the public health infrastructure, the mortality rate and property loss will be high. A lack of the public health assessment of infectious disease risks after the 2010 Haiti earthquake led to a cholera outbreak in mid-October, 2010, causing 4835 deaths [15]. A large-scale population migration caused by a natural or manmade disaster will in turn lead to various diseases caused by food shortage, water contamination, crowded shelters, and other hygienic problems. Therefore, disaster epidemiology is of particular importance in planning for, responding to, and recovering from disasters, especially in developing countries.

## Discussion

In developed countries, such as the U.S., there are effective measures for disaster preparedness and management. hospital HVAs have been required for more than 30 years, while community HVAs have more recently been required as part of emergency management and public health agency planning. However, in other countries, especially developing and under-developed countries such as China, where population vulnerability is high, there is a lack of effective planning policies. It is of critical importance for these countries to learn from the experiences of other countries and to implement essential procedures. In China, hospital HVAs should be set up first. To carry out the HVA in its entirety, designated departments and personnel should be appointed to oversee HVA activities. Later, collaborations between communities, hospitals, and other entities are needed to establish community HVAs. Here, we proposed the following recommendations, which are already in practice:

1. Conduct an annual HVA in each hospital and other health care faculty, with funding for and designated staff member(s) assigned to the HVA activity. Periodic training will ensure that the most up-to-date information is available to all participants.
2. Retrospective and prospective assessments of possible hazardous events are fundamental. Efforts should be made to establish reliable resources and obtain accurate information. In addition to those required by federal authorities, local priorities need to be considered.
3. The use of a standardized methodology for collecting information, analyzing data, and interpreting results will ensure the consistency and comparability of the HVA across hospitals and health care institutions. Detailed meeting minutes should be recorded during the decision-making process, including minority opinions and overall results.
4. The availability of mental health care for the vulnerable, disaster-affected population should be increased to meet the demand. Conditions include, but not limited to, schizophrenia, dementia, post-traumatic stress disorder and major depressive disorder, and anxiety or generalized anxiety disorder, as well as general mental health problems such as addictions, aggression, and sleep disorders [29].
5. The results of the HVA process should be distributed both within and outside of the institution.

## Conclusions

Natural disasters, such as floods or cyclones, will become more frequent. The probability of manmade or technological disasters is also increasing. The challenge that lies ahead is immense. A standardized and comprehensive HVA can help health care facilities identify and stratify potential hazards. The ultimate goal of an HVA is to establish a robust emergency management program. It identifies key positions and their responsibilities; prioritize tasks (immediate, intermediate and extended); sets plans for training, drilling, and exercising; and guides planning, mitigation and recovery projects [25]. The development, implementation and maintenance of a robust disaster preparedness plan needs great collaboration and synergy between different operational agencies and academic and training institutions. It is also important to merge academic advancement and expertise with practical field application.

## Abbreviations

DEA: data envelopment analysis
FEMA: Federal Emergency Management Agency
GIS: geographic information system
HVA: hazard vulnerability assessment
PPC: projection pursuit cluster
PTSD: post-traumatic stress disorder
